# Female Bias in *Rhox6* and *9* Regulation by the Histone Demethylase KDM6A

**DOI:** 10.1371/journal.pgen.1003489

**Published:** 2013-05-02

**Authors:** Joel B. Berletch, Xinxian Deng, Di Kim Nguyen, Christine M. Disteche

**Affiliations:** 1 Department of Pathology, School of Medicine, University of Washington, Seattle, Washington, United States of America; 2 Department of Medicine, School of Medicine, University of Washington, Seattle, Washington, United States of America; University of Pennsylvania, United States of America

## Abstract

The *Rho*x cluster on the mouse X chromosome contains reproduction-related homeobox genes expressed in a sexually dimorphic manner. We report that two members of the *Rhox* cluster, *Rhox6* and *9*, are regulated by de-methylation of histone H3 at lysine 27 by KDM6A, a histone demethylase with female-biased expression. Consistent with other homeobox genes, *Rhox6* and *9* are in bivalent domains prior to embryonic stem cell differentiation and thus poised for activation. In female mouse ES cells, KDM6A is specifically recruited to *Rhox6* and *9* for gene activation, a process inhibited by *Kdm6a* knockdown in a dose-dependent manner. In contrast, KDM6A occupancy at *Rhox6* and *9* is low in male ES cells and knockdown has no effect on expression. In mouse ovary where *Rhox6* and *9* remain highly expressed, KDM6A occupancy strongly correlates with expression. Our study implicates *Kdm6a*, a gene that escapes X inactivation, in the regulation of genes important in reproduction, suggesting that KDM6A may play a role in the etiology of developmental and reproduction-related effects of X chromosome anomalies.

## Author Summary

Homeobox (*HOX*) genes are known to be under epigenetic control during development. Here, we report that two mouse X-linked homeobox genes implicated in reproduction, *Rhox6* and *9*, are activated by the histone demethylase KDM6A that removes methylation at lysine 27 of histone H3. *Kdm6a* is one in a small group of genes that escape X inactivation in mice and humans, and thus has female-biased expression. We found that knockdown of *Kdm6a* affects *Rhox6* and *9* expression specifically in female ES cells. We also demonstrate that high expression of *Rhox6* and *9* in mouse ovary is associated with recruitment of KDM6A to these genes, consistent with a role in a female-specific organ. Furthermore, we demonstrate paternal imprinting of *Rhox6* and *9* in mouse ovary. The findings herein help to understand sex bias in the regulation of reproductive homeobox genes during early development and in ovary. Our findings provide clues into the sex-specific roles played by genes that escape from X inactivation, which may contribute to developmental defects and ovarian dysfunction in individuals with X chromosome abnormalities.

## Introduction

Homeobox (*HOX*) genes are known for their ability to regulate embryogenesis and guide tissue differentiation. These genes encode transcription factors that specify cell identity and regulate many embryonic programs including axis formation, limb development, and organogenesis [1]. Control of *HOX* gene expression via epigenetic modifications that include DNA methylation and histone modifications is critical to this process. Notably, tri-methylation of lysine residue 27 of histone H3 (H3K27me3) plays a major role in repression of *HOX* genes [2]. The histone demethylase KDM6A (also known as UTX) removes H3K27me3 from *HOX* genes to restore their activity [3]. KDM6A contains a tetratricopeptide motif predicted to mediate protein-protein interactions [4], and is a member of a stable multi-protein complex that not only de-methylates H3K27me3 but also methylates lysine 4 at histone H3 to facilitate gene expression [5], [6]. Different protein partners modulate KDM6A recruitment to specific chromatin regions since ectopic *KDM6A* expression does not result in significant reduction of genome-wide H3K27me3 levels but rather targets specific genes [3], [7], [8], [9]. For example, KDM6A regulates muscle-specific genes during myogenesis and is necessary for proper cardiac cell differentiation [10], [11]. KDM6A mutations have been discovered in patients with Kabuki syndrome, a rare syndrome associated with distinct facial features, intellectual disability, growth retardation, and skeletal anomalies [12], [13]. Recent studies have also implicated *KDM6A* as a candidate tumor suppressor gene whereby ectopic expression leads to enhanced expression of the *RB* (retinoblastoma) and *RBL2* (retinoblastoma-like 2) genes [14]. *KDM6A* inactivating mutations have been discovered in acute promyelocytic leukemia and multiple other cancer types [15], [16], [17].

A large set of homeobox genes clustered on the X chromosome has been implicated in male and female reproduction. In mouse, this cluster called *Rhox* (reproductive homeobox X-linked) contains 33 adjacent genes organized into three sub-clusters: α, β, and γ [18]. The *Rhox* cluster evolved at a rapid pace in mammals: the rat cluster contains 11 genes, and the human cluster, only 3 genes. In mouse, members of each paralog family have nearly identical sequences and are thus considered to be functional, although few members have been studied in detail [18]. *Rhox* genes are selectively expressed in male and female reproductive tissues, including testis, ovary, and placenta [19]. Similar to other homeobox genes, *Rhox* genes are also expressed during early embryonic development [19], [20], [21], [22], [23]. Little is known about the biological significance of individual paralogs.

Epigenetic regulation of the *Rhox* gene cluster has been mainly focused on DNA methylation and histone H1 control in placenta and during embryonic development [24], [25]. It is unknown whether other histone modifications control *Rhox* expression and what histone modifiers might be responsible. An important contender is KDM6A, which is known to regulate the *HOX* cluster [3]. Interestingly, KDM6A is encoded by an X-linked gene that escapes X inactivation in somatic tissues of human and mouse [26], [27], [28]. Expression of most X-linked genes in somatic tissues is equalized between males (XY) and females (XX) by random silencing of one X chromosome in early development [29]. Genes that escape X inactivation represent exceptional genes with higher expression in females versus males, suggesting that they may be important for female-specific functions [29], [30], [31].

To explore the potential role of KDM6A in the sex-specific regulation of *Rhox* genes, chromatin analyses were done to follow KDM6A recruitment to the *Rhox* cluster in male and female ES cells. We focused on *Rhox6* and *9*, two members of the *Rhox* cluster we discovered to be most affected by *Kdm6a* knockdown. KDM6A was specifically recruited to *Rhox6* and *9* in female but not male ES cells, resulting in removal of the repressive histone mark H3K27me3 and in increased expression. KDM6A was also bound to *Rhox6* and *9* in ovary where these genes are highly expressed. We conclude that KDM6A is important for removal of a repressive histone mark at the bivalent promoters of *Rhox6* and *9* to facilitate their expression in female ES cells and in ovary.

## Results

### *Rhox6* and *9* and *Kdm6a* are expressed in a sexually dimorphic manner

**Figure 1:**
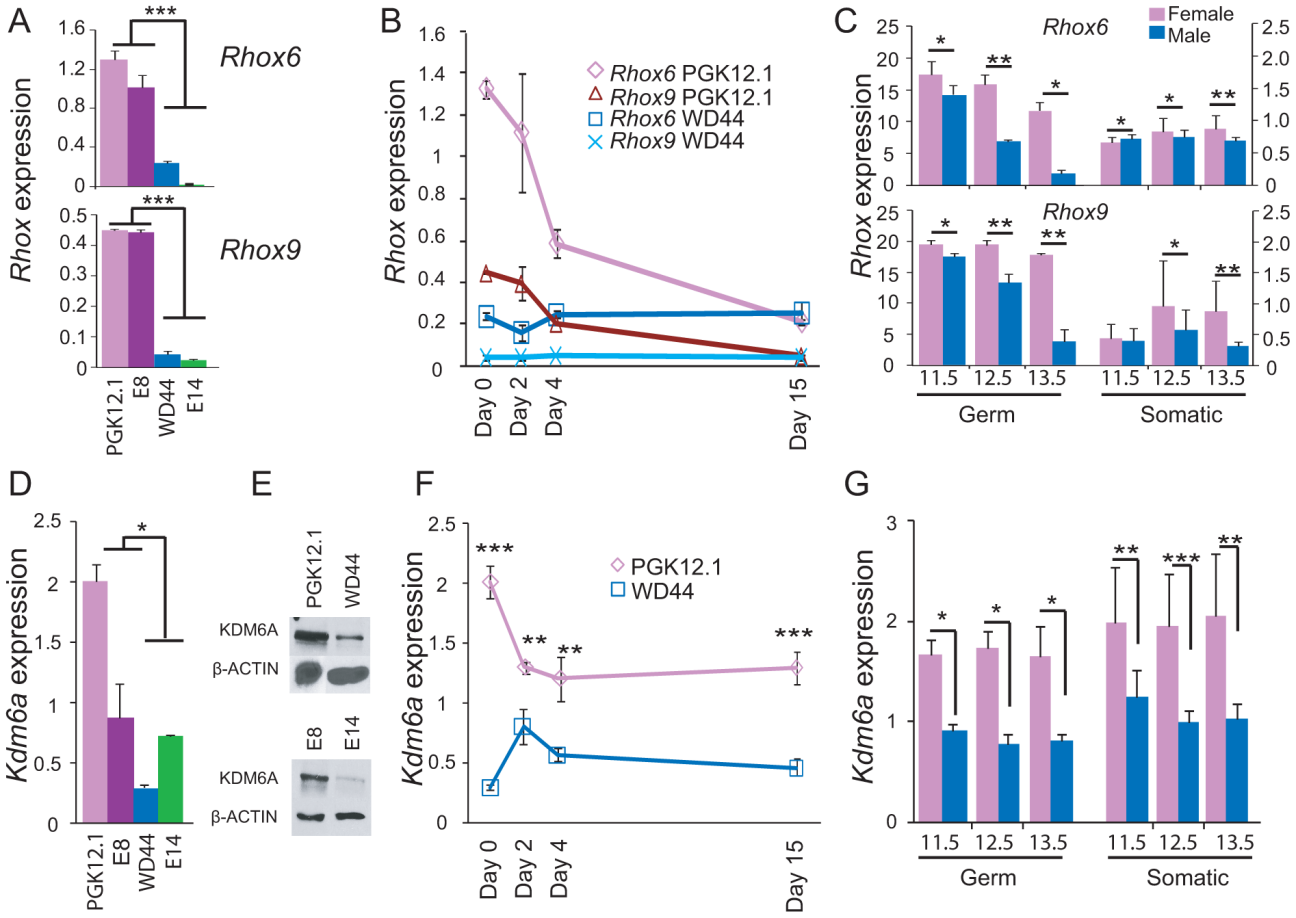
Sexual dimorphism of *Rhox6* and *9* and *Kdm6a* expression in ES cells and embryos. (A) *Rhox6* and *9* expression measured by qRT-PCR is higher in female (PGK12.1 and E8) than male (WD44 and E14) undifferentiated ES cells (***p<0.0001). Gene expression was normalized to *18s* levels. (B) *Rhox6* and *9* expression measured by qRT-PCR in female PGK12.1 ES cells and in male WD44 ES cells during ES cell differentiation. Gene expression was normalized to *18s* levels. (C) Re-analyses of published expression array data in germ cells and somatic cells in sexed embryos (11.5–13.5 dpc) shows higher *Rhox6* and *9* expression in female than male embryos (*p<0.05, **p<0.001) (see also Figure S2A). Endothelial, mesenchymal, and follicle cells were analyzed together as somatic cells (12 samples total). Values were normalized to the array mean. (D) *Kdm6a* expression measured by qRT-PCR is higher in female (PGK12.1 and E8) than male (WD44 and E14) undifferentiated ES cells (*p<0.05). Gene expression was normalized to *18s* levels. (E) Western blot analysis confirms higher protein levels in female (PGK12.1 and E8) versus male (WD44 and E14) ES cells. β-ACTIN is used as a control. (F) *Kdm6a* expression measured by qRT-PCR in female PGK12.1 ES cells and in male WD44 ES cells is higher in undifferentiated female than male ES cells throughout differentiation (**p<0.001, ***p<0.0001). Gene expression was normalized to *18s* levels. (G) Re-analyses of published expression array data in germ cells and somatic cells in sexed embryos (11.5–13.5 dpc) shows higher *Kdm6a* expression in female than male embryos (*p<0.05, **p<0.001, ***p<0.0001). Endothelial, mesenchymal, and follicle cells were analyzed together as somatic cells (12 samples total). was higher. Values were normalized to the array mean.

*Rhox6* and *9* expression levels were significantly higher in undifferentiated female versus male ES cells (Figure 1A). Expression was measured using quantitative RT-PCR (qRT-PCR) in two female (PGK12.1 and E8) and two male (WD44 and E14) ES cell lines before and after differentiation. ES cell differentiation and embryoid body formation were induced by removal of LIF (leukemia inhibitory factor). *Rhox6* and *9* primers were verified to be gene-specific by cDNA sequencing (Figure S1). Sex-specific differences persisted to day 2 of ES cell differentiation (Figure 1B). Analyses of sexed 8-cell pre-implantation embryos confirmed higher female than male expression of *Rhox6* and *9* in early development in vivo (Figure S2A). Furthermore, re-analyses of published microarray expression data [32] revealed a female bias in *Rhox6* and *9* expression at later embryonic stages (11.5–13.5 dpc) in both germ cells (at all stages) and somatic cells (at 12.5–13.5 dpc) (Figure 1C). Note that expression was much higher in germ cells compared to somatic cells.

**Figure 2:**
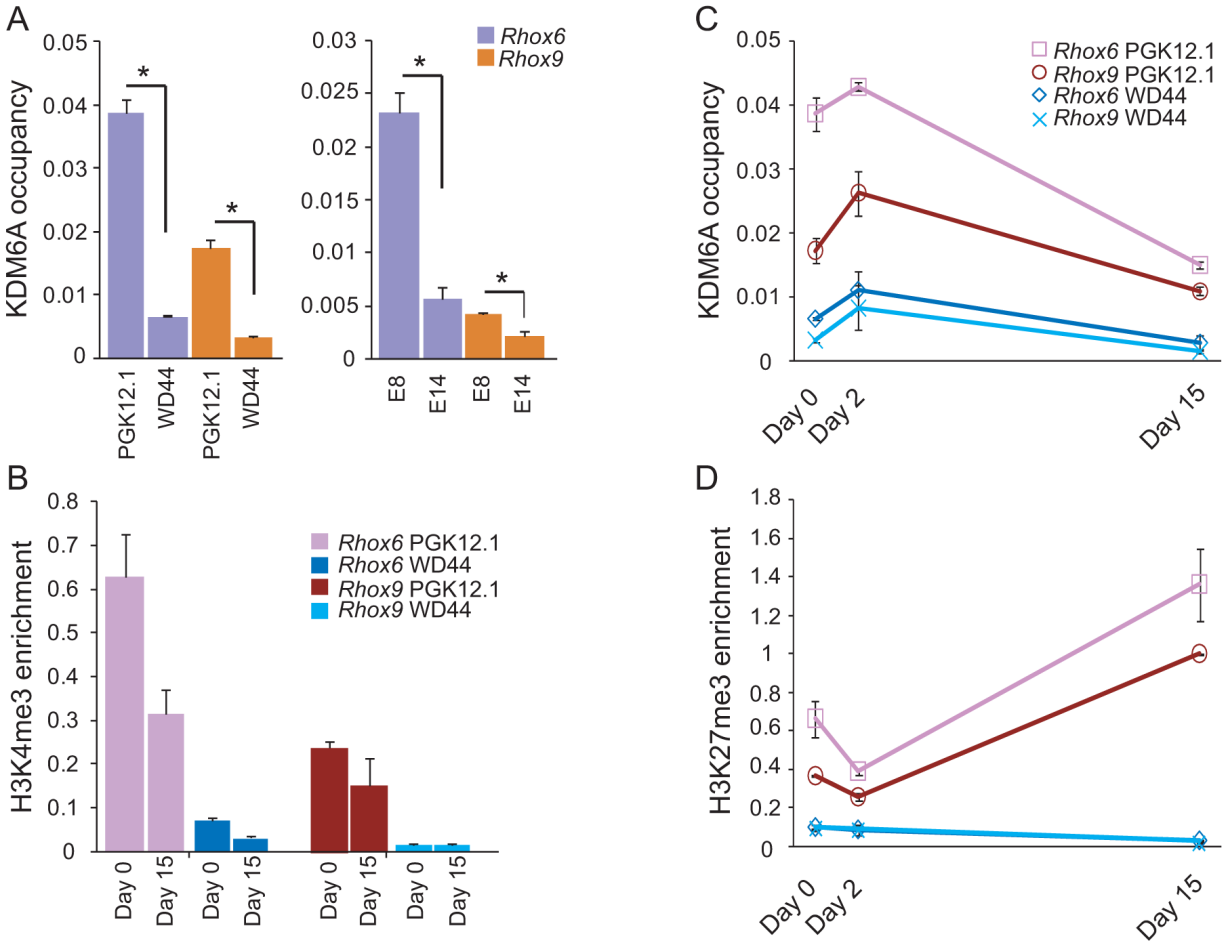
KDM6A is preferentially recruited to *Rhox6* and *9* in female ES cells. (A) ChIP-qPCR analysis of KDM6A occupancy at the 5′ end of *Rhox6* and *9* is higher in female (PGK12.1 and E8) than male (WD44 and E14) undifferentiated ES cells (*p<0.05). (B) H3K4me3 enrichment during differentiation of female PGK12.1 and male WD44 ES cells shows lower levels in male ES cells and a decrease of between day 0 and 15 in agreement with gene silencing after differentiation of these ES cells (see also Figure 1). (C) KDM6A occupancy at the 5′ end of *Rhox6* and *9* during differentiation of female PGK12.1 ES cells and male WD44 ES cells. (D) H3K27me3 levels at the 5′ end of *Rhox6* and *9* mirror KDM6A occupancy changes. The increase at day 15 is due to X inactivation in female PGK12.1 ES cells (see also Figures S2B, S4, and S6). Average enrichment/occupancy for two separate ChIP experiments is shown as ChIP/input (A, B, C).

A female bias in *Rhox6* and *9* expression in ES cells was unexpected because these genes are solely expressed from the maternal allele due to paternal imprinting [25]. Thus, the significantly higher expression we observed in undifferentiated female versus male ES cells (>6-fold for *Rhox6* and >10-fold for *Rhox9*, respectively) must be due to another factor (Figure 1A). Interestingly, levels of the histone demethylase KDM6A known to play a role in HOX gene regulation were approximately two-fold higher in female compared to male ES cells as measured by qRT-PCR and western blot analyses (Figure 1D, 1E). This sex bias initially due to the presence of two active X chromosomes in undifferentiated female ES cells [33], [34] persisted throughout differentiation and at later embryonic stages, as expected for a gene that escapes X inactivation (Figure 1F, 1G). To determine whether KDM6A was involved in the sex-specific regulation of *Rhox6* and *9* we measured occupancy using chromatin immunoprecipitation (ChIP) in male and female ES cells. KDM6A occupancy at the 5′ end of *Rhox6* and *9* was greater in undifferentiated female than male ES cells as measured both by quantitative PCR (ChIP-qPCR) and by array analysis (ChIP-chip) (Figure 2A and Figure S2B). At day 2 of differentiation the female bias in KDM6A occupancy persisted, but following differentiation (day 15) KDM6A occupancy decreased (Figure 2C and S2B). These results are in agreement with the observed timing of changes in *Rhox6* and *9* expression (Figure 1B). Furthermore, we observed corresponding changes in levels of H3K27me3, the histone modification removed by KDM6A. By ChIP-qPCR H3K27me3 enrichment mirrored changes in KDM6A occupancy at *Rhox6* and *9* in female PGK12.1 ES cells during differentiation (Figure 2D). Quantitative analysis of H3K4me3 enrichment at *Rhox6* and *Rhox9* promoters revealed higher enrichment in female than male ES cells, as well as a decrease during differentiation correlating with expression changes (Figure 2B, Figure 1A and 1B).

During differentiation X inactivation initiates in female PGK12.1 ES cells, as confirmed by increased *Xist* expression and by the appearance of an *Xist* cloud detected by RNA-FISH in interphase nuclei of cells at day 15 (Figure S3) [35]. Concomitantly, H3K27me3 enrichment at *Rhox6* and *9* increased almost 2–3-fold between day 0–2 and day 15 (Figure 2D). This increase was observed over the entire *Rhox* cluster, suggesting that the cluster is subject to silencing possibly by X inactivation (Figure S4) (see below) [29]. In male ES cells, H3K27me3 levels were very low at *Rhox6* and *9* at all time points (Figure 2D). Taken together, our data indicate that KDM6A is specifically recruited to *Rhox6* and *9* in undifferentiated female ES cells, which results in a 6–10-fold higher expression compared to male ES cells.

### KDM6A regulates *Rhox6* and *9* expression in female but not male ES cells

**Figure 3:**
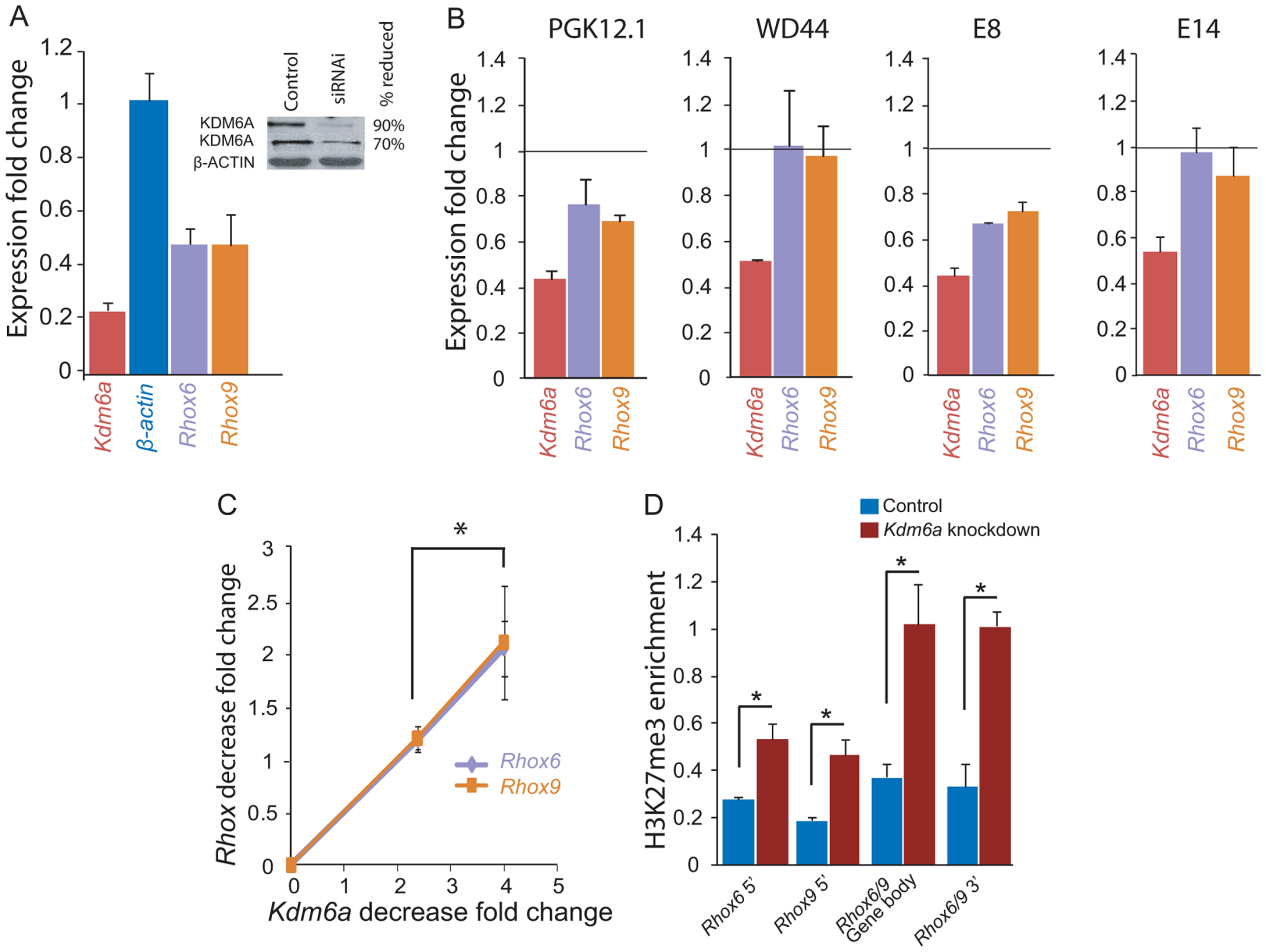
*Kdm6a* knockdown causes a female-specific decrease in *Rhox6* and *9* expression in ES cells. (A) Quantitative RT-PCR after *Kdm6a* knockdown in female PGK12.1 ES cells shows a 75% and a 52% decrease in *Kdm6a* and *Rhox6* and *9* expression, respectively. Expression is shown relative to control levels obtained with scrambled siRNA. Control gene *β-actin* levels are set to 1. The inset shows a western blot using two different KDM6A antibodies, which confirms a ∼70–90% reduction in protein levels. β-ACTIN is used as a control. (B) *Kdm6a* knockdown results in a decrease in *Rhox6* and *9* expression in female (PGK12.1 and E8) but not male (WD44 and E14) undifferentiated ES cells. Expression measured by array analysis (PGK12.1 and WD44) and qRT-PCR (E8 and E14) is shown as fold change compared to the control gene *β-actin*. Array results are from four independent experiments and qRT-PCR results are from three independent experiments. (C) The decrease in *Rhox6* and *9* expression correlates with the level of *Kdm6a* knockdown in a dose dependent manner in PGK12.1 ES cells (*p<0.05). Fold changes in levels of *Rhox6* and *9* measured by expression arrays are shown relative to fold changes in *Kdm6a* levels. (D) *Kdm6a* knockdown results in a significant (*p<0.05) increase in H3K27me3 enrichment at *Rhox6* and *9* 5′end, gene body, and 3′end. ChIP-qPCR of H3K27me3 enrichment in PGK12.1 ES cells treated with control scrambled siRNA and *Kdm6a* specific siRNA. Average enrichment for two separate ChIP experiments is shown as ChIP/input.

To directly assess the role of KDM6A in regulation of the *Rhox* cluster we performed knockdowns in two female and two male ES cell lines by RNAi. Using a pool of siRNAs to target multiple regions of *Kdm6a* RNA, we achieved a 60–80% knockdown in ES cells as shown by qRT-PCR and expression array analyses (Figure 3A). Immunoblots using two different antibodies confirmed a dramatic reduction (70–90%) in the amount of KDM6A protein after 48 h of knockdown (Figure 3A). Specificity of the siRNAs was confirmed using two individual siRNAs, each resulting in a ∼60% knockdown (Figure S5A).

*Kdm6a* knockdown caused a significant reduction in *Rhox6* and *9* expression in the two female (PGK12.1 and E8) but not in the male (WD44 and E14) ES cell lines, indicating that the regulation of these genes by KDM6A is female-specific (Figure 3B). *Rhox6* and *9* expression levels measured by qRT-PCR and by expression array analyses were diminished by 30–50% after *Kdm6a* knockdown whereas the control gene *β-actin* did not change (Figure 3A, 3B). By expression array analyses we found that among the *Rhox* genes, *Rhox6* and *9* exhibited the highest expression decrease (>1.25 fold) (Table S1). The lesser decrease measured by expression arrays versus qRT-PCR can be attributed to the different methodologies; qRT-PCR was done using primers designed to be specific for either *Rhox6* or *Rhox9* (Figure S1), whereas expression changes measured by arrays may be dampened by cross-hybridization due to high sequence similarity between the genes. Importantly, *Rhox6* and *9* expression depended on the amount of *Kdm6a* knockdown in a dose-sensitive manner, consistent with a sex-specific dosage effect (Figure 3C). Note that *Rhox5* also showed a significant decrease after *Kdm6a* knockdown but its analysis was not pursued at this time. As expected, KDM6A occupancy was reduced at the 5′ end and gene body of *Rhox6* and *9* after knockdown in female ES cells (Figure S5B). Conversely, H3K27me3 levels were significantly increased at the 5′ end, gene body, and 3′end of *Rhox6* and *9*, while there was no significant change in levels of H3K4me3 (Figure 3D and Figure S5C). Genes associated with pluripotency (e.g. *Cd9, Nanog, Pou5f1, Stat3, Sox2*) were not affected by *Kdm6a* knockdown in either female or male ES cells indicating no induction of differentiation (Figure S5D). We conclude that KDM6A plays a critical and dose-dependent role in regulating *Rhox6* and *9* expression in female but not male ES cells.

### *Rhox6* and *9* are bivalently marked in undifferentiated ES cells

**Figure 4:**
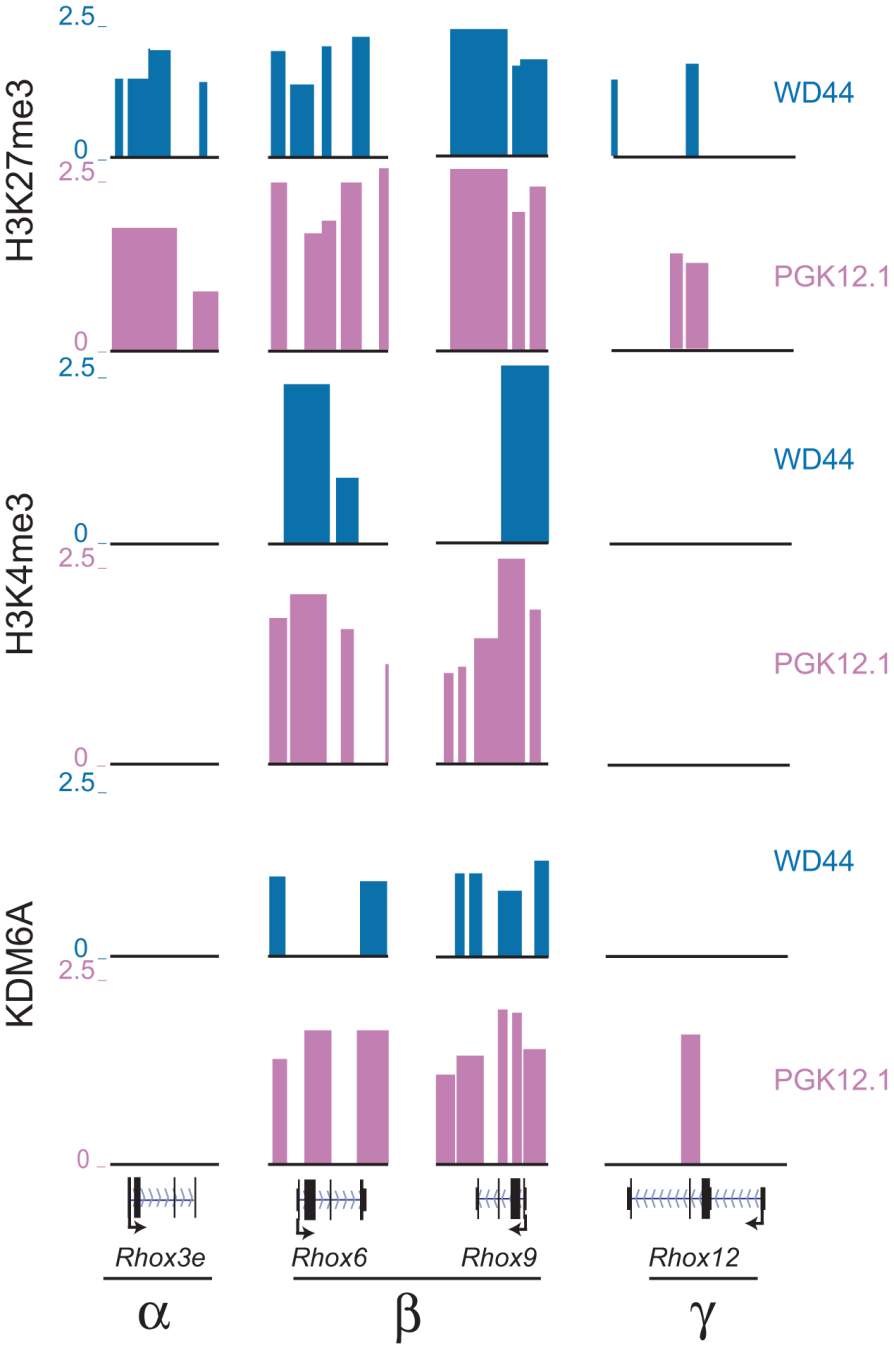
*Rhox6* and *9* are bivalent and preferentially occupied by KDM6A in female ES cells. H3K27me3, H3K4me3 and KDM6A enrichment profiles in undifferentiated female PGK12.1 (pink) and male WD44 ES (blue) cells at representative genes from each *Rhox* subcluster (α, β, and γ) demonstrate that only *Rhox6* and *9* are highly enriched with both histone modifications and are bound by KDM6A (see also Figure S6). *Rhox3e* (α cluster) is enriched in H3K27me3 but not H3K4me3 or KDM6A, and *Rhox12* (γ cluster) shows little enrichment for the proteins analyzed. Significant enrichment peaks based on Nimblescan analysis (FDR score <.05) are shown. Data uploaded to UCSC genome browser (NCBI36/mm8).

Chromatin domains that contain both activating and inactivating histone marks in undifferentiated ES cells have been termed bivalent and are thought to be poised for activation during development [36], [37]. Notably, bivalent genes include homeobox genes, such as *HOX* genes, suggesting that *Rhox* genes are also candidates for bivalency. ChIP-chip profiles in both female and male undifferentiated ES cells demonstrated that both *Rhox6* and *9* in cluster β were enriched in H3K27me3 and H3K4me3, indicating that these genes are bivalent and thus poised for activity during development (Figure 4 and Figure S6). Quantitative measurements showed higher H3K4me3 and H3K27me3 levels in female versus male ES cells (Figure 2B, 2D). H3K4me3 levels decreased and H3K27 levels increased between day 0 and 15 of differentiation in female ES cells, consistent with a decrease in *Rhox6* and *9* expression (Figure 2B, 2D). In contrast, levels remained low in male ES cells. KDM6A binding being clearly female-biased would explain the female bias in gene expression at day 0–2 of differentiation, as described above (Figure 1 and Figure 2).

Other *Rhox* genes within the α- and γ-clusters did not appear to be bivalent but rather were enriched with the repressive mark H3K27me3, with no significant peaks of enrichment for the active mark H3K4me3, suggesting that these genes are mostly contained in a silenced chromatin domain in both female and male undifferentiated ES cells (Figure 4 and Figure S6). Inspection of representative genes from each cluster, *Rhox6* and *9* in cluster β, *Rhox3e* in cluster α, and *Rhox12* in cluster γ, confirmed these findings (Figure 4). Note that while *Rhox1* and *7* also appeared bivalent at day 0, KDM6A was absent at their promoter, which may account for their low expression (Table S1).

### *Rhox6* and *9* are bound with KDM6A and highly expressed in ovary

**Figure 5:**
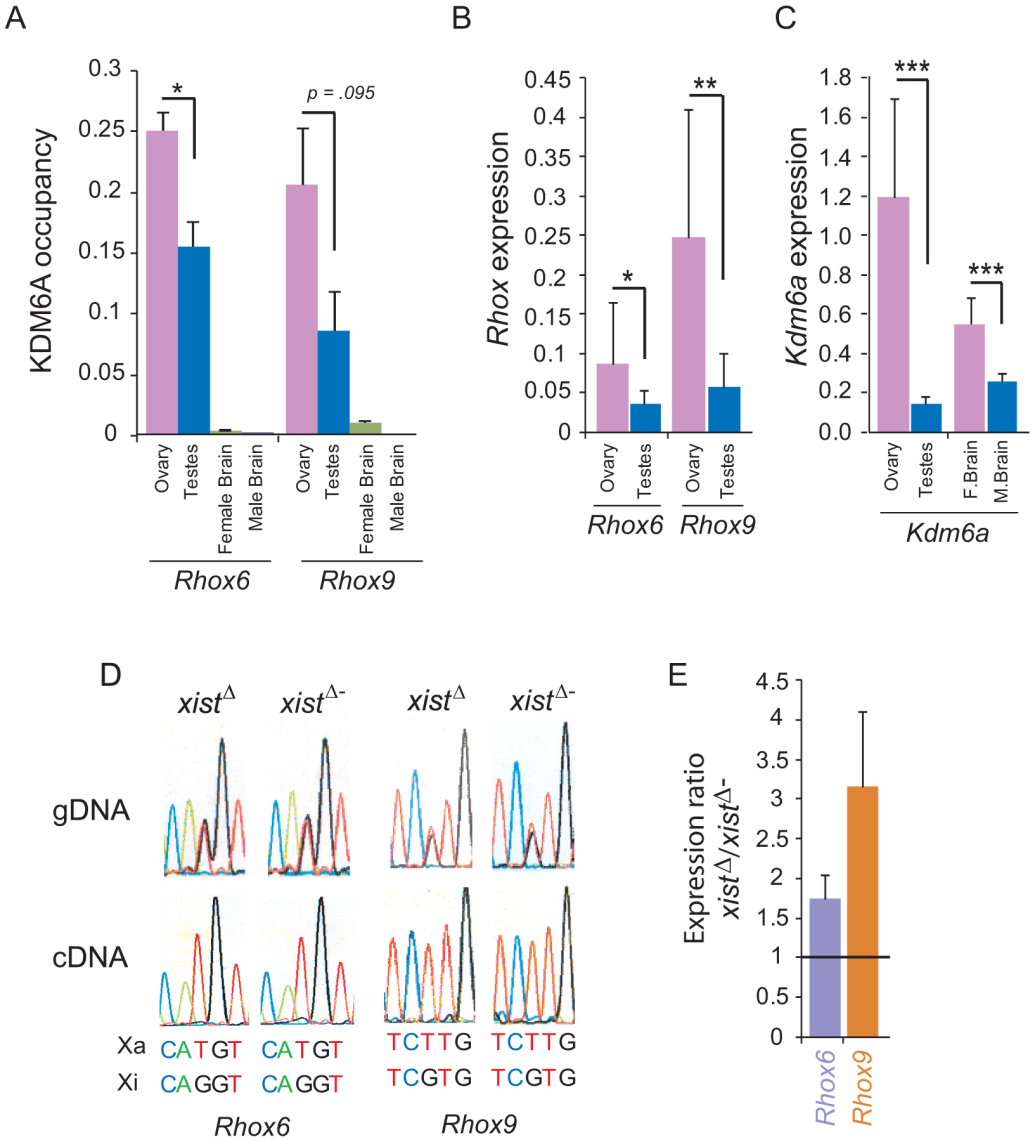
*Rhox6* and *9* expression and KDM6A occupancy are high in ovary where the genes are imprinted. (A) *Rhox6* and *9* have significantly higher expression in mouse ovary than in testis, based on re-analyses of published expression array data for 14 testis and 12 ovary specimens (*p<0.05, **p<0.001). Expression normalized to array mean (see also Figure 1C). (B) KDM6A occupancy measured by ChIP-qPCR at the 5′end of *Rhox6* and *9* is higher in ovary than in testis, and is very low to undetectable in brain where these genes are not expressed [19]. Occupancy levels were normalized to input fractions. (C) *Kdm6a* has high expression in female tissues especially ovary based on analyses of published expression array data (***p<0.0001) (see also Figure 1F). (D) *Rhox6* and *9* are expressed from the maternal allele only in ovary because of imprinting. DNA sequence chromatograms of gDNA and RT-PCR (cDNA) products derived from ovary from female F1 mice obtained by mating *M. spretus* males with C57BL/6J females with or without an *Xist* mutation (*Xist^Δ^* and *Xist^Δ−^*). SNPs to distinguish *Rhox6* and *9* alleles on the active X (Xa) and on inactive X (Xi) are indicated below. In ovary from both *Xist^Δ^* and *Xist^Δ−^* mice the gDNA shows heterozygosity at the SNPs while the cDNA shows only the maternal allele, consistent with paternal imprinting. (E) By qRT-PCR *Rhox6* and *9* are more highly expressed in ovary from *Xist^Δ^* mice in which the maternal X chromosome is expressed in all cells, compared to *Xist^Δ−^* mice in which there is random X inactivation (1.7-fold and 3-fold, respectively), suggesting that *Rhox6* and *9* are silenced by X inactivation. Values represent the expression ratio between *Xist^Δ^* and *Xist^Δ−^* ovaries.

Re-analyses of published expression array data confirmed that *Rhox6* and *9* and *Kdm6a* are expressed at a higher level in ovary than in testis (Figure 5A) [18], [19]. This is consistent with measurements of expression in embryos, in which female germ cells have much higher expression than male germ cells (Figure 1C). As expected for a gene that escapes X inactivation, *Kdm6a* expression was higher in all female tissues examined in comparison to male tissues, including brain and sexual organs, as well as somatic and germ cells from embryos (Figure 5C and Figure 1G). To assess the *in vivo* binding of KDM6A to *Rhox6* and *9* in reproductive tissues, chromatin extracted from adult mouse ovaries and testes was subjected to ChIP-qPCR. KDM6A occupancy was high at the promoters of *Rhox6* and *9* in mouse ovary, consistent with high expression in this organ (Figure 5A, 5B) [38], [39]. In mouse testis where *Rhox6* and *9* expression is lower, KDM6A was still bound but to a lesser extent (38% of occupancy in ovary), reflecting lower expression (Figure 5A, 5B). In mouse brain where the genes are not expressed [19], KDM6A occupancy was almost undetectable in females and completely undetectable in males (Figure 5B). Taken together, these data indicate that KDM6A occupancy is associated with *Rhox6* and *9* expression in reproductive tissues, more significantly in females than in males.

### *Rhox6* and *9* are paternally imprinted in ovary

To determine the allele-specific expression of *Rhox6* and *9* in ovary we employed F1 mice derived from crosses between C57BL/6J females with (*Xist^Δ^*) or without (*Xist^Δ−^*) an *Xist* mutation and *Mus spretus* males. In F1 animals that carry the mutant *Xist* (*Xist^Δ^*), X inactivation is completely skewed towards the *M. spretus* X chromosome. SNPs between the mouse species were used to distinguish alleles after RT-PCR and Sanger sequencing. In F1 mice with (*Xist^Δ^*) or without (*Xist^Δ−^*) the *Xist* mutation expression of *Rhox6* and *9* was exclusively from the maternal C57BL/6J allele, with no evidence of the *M. spretus* allele, consistent with imprinting of the paternal allele (Figure 5D). This is similar to what has been reported in mouse ES cells and placenta [25]. Control genomic DNA amplification confirmed the presence of the SNPs in the F1 mice (Figure 5D). Our results suggest that imprinting has taken place in the germ cells from adult ovary, as we did not observe any evidence of paternal allele expression. By qRT-PCR *Rhox6* and *9* expression was higher (1.7-fold and 3-fold, respectively) in ovaries of F1 mice carrying the *Xist* mutation (*Xist^Δ^*) in which the maternal allele is expressed in all cells (due to skewing of X inactivation), compared to ovaries from non-mutant F1 mice (*Xist^Δ−^*) in which the maternal allele is expressed in half of the cells (due to random X inactivation) (Figure 5E). The X inactivation effect on *Rhox6* and *9* expression would only be pertinent in somatic cells, but not in germ cells in which the inactive X chromosome is reactivated. This complicates interpretation of our data because expression was measured in whole ovary containing both germ cells with very high *Rhox6* and *9* expression and supporting somatic cells with lower expression (Figure 1C). Additional studies in germ cells and somatic cells of the ovary are needed to fully understand the developmental regulation of *Rhox6* and *9* in this organ. Nonetheless, we conclude that female biased expression of *Rhox6* and *9* in ovary is not due to bi-allelic expression in this tissue but rather to recruitment of KDM6A to activate *Rhox6* and *9* on the active maternal X chromosome.

## Discussion

*Rhox* genes represent a set of X-linked homeobox genes specifically expressed in organs and cell types implicated in sexual development and reproduction [19], [20], [40], [41]. Here, we provide functional evidence identifying KDM6A, an enzyme that removes methylation at lysine 27 of histone H3, as an important regulator of a specific subset of *Rhox* genes, *Rhox6* and *9*, in female ES cells and in ovary. Interestingly, KDM6A is encoded by an X-linked gene that escapes X inactivation and has higher expression in females, which may indirectly facilitate its sex-specific role in enhancing *Rhox6* and *9* expression [26], [27], [28]. Our knockdown experiments clearly support an important role for KDM6A in regulating *Rhox6* and *9* in female but not male ES cells.

The 6–10 fold female bias in *Rhox6* and *9* expression we measured in undifferentiated ES cells cannot be explained by the presence of two active X chromosomes in female ES cells prior to X inactivation since *Rhox6* and *9* are paternally imprinted in these cells [25]. Rather, the female enhanced expression results from the specific recruitment of KDM6A at those genes to facilitate the transition from repressive to active histone modifications and to increase expression. A similar mechanism explains the female bias in *Rhox6* and *9* expression in ovary where we demonstrate that the genes are imprinted as well. KDM6A is a member of a multi-protein complex that not only de-methylates H3K27me3 but also methylates lysine 4 at histone H3 to facilitate gene expression [5], [6]. KDM6A counterbalances polycomb activity by regulating H3K27me3 levels [9], which would help maintain *Rhox6* and *9* expression in undifferentiated female ES cells and ovary. In differentiated female ES cells KDM6A occupancy decreases, which mirrors the accumulation of H3K27me3 at *Rhox6* and *9*, consistent with low expression in most somatic cell types as well as with the heavy DNA methylation reported for these genes during development [24]. Our analyses of 8-cell embryos suggest that a female bias in *Rhox6* and *9* expression is already present at this early stage, prior to gonadal development. Somatic and germ cells from developing embryos still show a female bias in *Rhox6* and 9 expression at later stages (11.5–13.5dpc) including those coincident with gonad differentiation, which confirms a previous study [20]. However, detailed analyses of sexed embryos at additional stages will be needed to fully follow developmental expression in multiple cell types.

Strikingly, within the *Rhox* cluster only *Rhox6* and *9* show a marked increase in KDM6A in female ES cells. Tellingly, these genes share a high degree of sequence similarity but differ from the other *Rhox* genes in other sub-clusters [19], suggesting that they may contain a sequence motif to specifically recruit KDM6A. The question arises of which other histone demethylases would remove H3K27me3 at other *Rhox* genes to facilitate their expression in specific tissues. We determined that KDM6B, which also removes methylation at lysine 27 of histone H3, has low expression in undifferentiated male and female ES cells (data not shown). However, its expression increases after differentiation in both male and female ES cells, pointing towards a potential role for this enzyme in regulating expression of some of the other *Rhox* genes at later stages of development [42]. KDM6A binds to the promoter, gene body, and 3′end of *Rhox6* and *9* in female ES cells, suggesting a mechanism of regulation at transcription initiation and elongation. Interestingly, recruitment of elongation factors to target genes has been demonstrated for KDM6B, in addition to its role in histone demethylation [43].

Epigenetic regulation of the *Rhox* cluster had been previously focused on DNA methylation [24], [44]. *Rhox5* whose expression peaks at day 9 after ES cell differentiation is repressed by DNA methylation at later stages, while it remains unmethylated and highly expressed in extra-embryonic tissues. Similarly, *Rhox6* and *9* are repressed following the establishment of CpG methylation by DNA methyltransferases DNMT3b and DNMT1 at their promoter regions in the embryo proper but not in extra-embryonic tissues [24]. *Rhox5* is the only gene together with *Xist* known to be expressed from the paternal X chromosome (maternally imprinted) at early embryonic stages (until e6.5); surprisingly, it is expressed from the maternal X (paternally imprinted) in extra-embryonic tissues, like *Rhox6* and *9* [18], [24], [25], [45]. Our results are consistent with paternal imprinting of *Rhox6* and *9* in mouse ovary, in agreement with other studies in placenta and ES cells [25].

We found that *Rhox6* and *9* are bivalently marked in undifferentiated ES cells as they are occupied by nucleosomes containing histone H3 methylated at both lysine 27 and lysine 4. Bivalent genes are usually silent while poised for expression [37]. However, *Rhox6* and *9* are in fact expressed in undifferentiated ES cells, probably due to a specific recruitment of KDM6A in a portion of cells in which levels of H3K27me3 would be decreased. Bivalent modifications result from a dynamic equilibrium of negative and positive chromatin marks controlled by histone modifying enzymes such as KDM6A. Our findings of a H3K27me3 increase at *Rhox6* and *9* after *Kdm6a* knockdown are in agreement with what has been reported for other bivalent genes and support a role for KDM6A in maintaining a balance between active and inactive marks at bivalent promoters [9]. Note that the extent of reduction in *Rhox6* and *9* expression we measured in female ES cells is comparable to that reported for another *HOX* gene, *HOXB1*, after *KDM6A* knockdown in human cells [8]. Many homeobox genes important for specification of cell types and organs contain bivalent domains, suggesting that bivalency is an important part of stem cell differentiation and development [46], [47]. Except for *Rhox6* and *9*, most other *Rhox* genes are not occupied by bivalent marks in undifferentiated ES cells, thus *Rhox6* and *9* may be specifically activated to influence lineage commitment. It will be interesting to determine which pathways and specific lineages are stimulated by RHOX6 and RHOX9 proteins. *Rhox6* has been implicated in the determination of the germ cell lineage [48]. So far, only *Rhox5* and *9* have been studied *in vivo*. Whereas *Rhox5*-null male mice exhibit increased germ cell apoptosis and sperm motility defects leading to sub-fertility, *Rhox9*-null male or female mice do not have any apparent phenotypes [19], [49]. It is possible that due to similarities in sequence, homeodomain, and expression patterns *Rhox6* compensates for the loss of *Rhox9* in these knockout mice [49], [50]. Additional evidence based on knockouts in mouse and rat epididymis, suggests that *Rhox5* may act as a master regulator of many of its paralogs [51].

Both *Rhox6* and *9* are highly expressed in ovary and to a lesser extent, in testis (this study) and [19]. An intriguing finding from our study is that KDM6A occupancy is high at *Rhox6* and *9* in ovary and thus may serve to keep these two *Rhox* genes active. KDM6A is also bound to *Rhox6* and *9* in testis, although at a lower level (1.7-fold and 2.5-fold lower in testis than in ovary, respectively), suggesting a threshold effect and/or another level of control in testis. Our knockdown experiments do indicate that KDM6A affects *Rhox6* and *9* expression in a dose-dependent manner. In embryonic gonads the majority of *Rhox* genes are already expressed in a sexually dimorphic manner from an early stage. Specifically, *Rhox6* and *9* are predominantly expressed in female versus male primordial germ cells at 12.5–15.5dpc (this study) and [19], [20], [38], [52]. In addition, *Rhox6* and *9* are also expressed in somatic cumulus cells in ovary [53], [54]. Our findings indicate that *Rhox6* and *9* are both imprinted on the paternal allele and subject to X inactivation. This implies the existence of a population of somatic cells without any *Rhox6* and *9* expression, suggesting that cumulus cells tolerate such mosaicism. In contrast, all germ cells would express *Rhox6* and *9* following X re-activation and subsequent imprinting of the paternal X chromosome. This is similar to what has been reported for some of the *Xlr* genes, a family of mouse genes also implicated in reproduction, some of which are also imprinted and subject to X inactivation [55].

In addition to removal of H3K27me3, KDM6A appears to have a demethylase-independent role in regulating chromatin structure [9], [56], [57]. Indeed, KDM6A and KDM6B regulate T-box family members through an interaction with SMARCA4-containing SWI/SNF complexes in T-cells [58]. Interestingly, *Kdm6a* knockout mice display a more severe phenotype at mid-gestation in female than male embryos [9], [59]. Thus, the Y-linked paralog *Uty* compensates for *Kdm6a* deficiency allowing survival of male embryos by a demethylase independent mechanism, since UTY does not have demethylase activity [9], [56], [59]. However, while some KDM6A-deficient male mice survive, most do not or are runted throughout adulthood, indicating that H3K27 demethylation remains an important function of KDM6A for survival and growth [56]. Additionally, histone demethylation appears to be the predominant mechanism required for activation of genes important in differentiation since mouse and human cells lacking KDM6A but retaining UTY fail to reprogram [60]. Furthermore, male primordial germ cells lacking KDM6A do not develop, as H3K27me3 levels are retained when compared to wild type [60]. Our knockdown experiments are consistent with a role for KDM6A in controlling levels of H3K27me3 and expression of *Rhox6* and *9* in female ES cells. However, we cannot rule out the contribution of a demethylase independent mechanism since we did not test for one in the context of *Rhox* expression control.

It remains to be determined whether levels of KDM6A are critical for proper ovarian function. It is interesting that female mice with a single X chromosome, which would have a lower dose of KDM6A due to haploinsufficiency for *Kdm6a*, a gene that escapes X inactivation, have reduced fertility [26], [61], [62]. Furthermore, XO female mouse embryos are developmentally retarded when compared to XX littermates at early mid-gestation [63]. In human, the presence of a single X chromosome causes Turner syndrome associated with severe developmental defects and ovarian dysgenesis [62]. It will be important to determine whether any of the human *RHOX* genes are also regulated by KDM6A. Mutations of *KDM6A* in human cause Kabuki syndrome, associated with growth retardation, unique facial features, and severe intellectual disability. Both males with point mutations and females with complete heterozygous deletions have been reported [12], [13]. The Kabuki phenotype, present in females who have one deleted *KDM6A* copy but absent in Turner syndrome females, may be due to partial silencing of the normal copy by X inactivation in Kabuki females, while Turner females would have one expressed copy in all cells. It would be interesting to examine ovaries in these patients.

In summary, our study provides the first evidence that *Rhox6* and *9* are regulated by the histone demethylase KDM6A in mouse ES cells and reproductive organs in a sex-specific manner. Our findings indicate that a gene that escapes X inactivation plays a sex-specific role in gene regulation in female ES cells and tissue. Higher female expression due to escape from X inactivation of *Kdm6a* may be favorable to *Rhox6* and *9* expression in ovary.

## Materials and Methods

### ES cell culture and differentiation and mouse tissue collection

Male ES cells WD44 (from C. Ware, University of Washington, US) and E14 *(BL6/Cast)* (from J. Gribnau, Erasmus MC Rotterdam, NL), and female ES cells PGK12.1 [64] and E8 *(BL6/Cast)* (from J. Gribnau, Erasmus MC Rotterdam, NL) were grown in high glucose DMEM media supplemented with 15% fetal bovine serum (FBS), 1% non-essential amino acids, 10 mg/ml APS, 0.1 mM 2-mercaptoethanol and 25 mM L-glutamine. ES cells were maintained in the presence of 1000 U/ml leukemia inhibitory factor (LIF) (Millipore) on a mono-layer of chemically inactivated mouse embryonic fibroblasts (MEF) and grown in a humidified incubator at 37°C and 5% CO_2_. Plates were enriched for ES cells by incubation on 1% gelatin coated dishes for 30 min to allow MEFs to attach, followed by transfer to fresh gelatin coated plates for overnight culture. Differentiation was achieved by removing LIF and culturing on non-adherent dishes to facilitate the formation of embryoid bodies. After 7 days, embryoid bodies were transferred to cell culture dishes for 8 days. Cells were harvested on days 0, 2, 4, and 15 after LIF removal. To follow X inactivation in PGK12.1 cells before and after differentiation, *Xist* expression was determined by RT-PCR and by RNA-FISH using standard protocols with a probe for *Xist* (Vysis) (Figure S3). Ovary, testis, and whole brain were collected from adult female and male C57BL/6J mice. Additionally, ovaries were collected from female F1 obtained by mating *M. spretus* males (Jackson Labs) with females that carry an *Xist* mutation (*Xist^Δ^*) (B6.Cg-Xist<tm5Sado>) (from T. Sado, Kyushu University, available from RIKEN) [65]. Female progeny were genotyped to verify inheritance of the mutant *Xist* allele. Female progeny with a *Xist^Δ^* fail to silence the BL6 X chromosome and thus have complete skewing of inactivation of the *spretus* X chromosome. All procedures involving animals were reviewed and approved by the University Institutional Animal Care and Use Committee (IACUC), and were performed in accordance with the Guiding Principles for the Care and Use of Laboratory Animals.

### *Kdm6a* siRNA knockdown

Stealth Select siRNAs (Invitrogen) were selected to target three different locations of the *Kdm6a* mRNA. The three siRNAs were transfected together or individually and oligonucleotides with no target were used as negative controls. For transfection, 5 µl of Lipofectamine RNAi Max Reagent (Invitrogen) mixed with 250 µl of Opti-MEM I Reduced Serum Medium (Invitrogen) containing 100 pmol of siRNAs were incubated for 30 min prior to addition to 6-well plates seeded with 1.5×10^5^ ES cells in 2 ml of DMEM supplemented as stated above. Cells were harvested after 48 h of RNAi treatment. Knockdown was confirmed by qRT-PCR, expression arrays, and Western blotting using standard procedures. Immunoblot analysis was done using a KDM6A/UTX antibody either from K.Ge (NIDDK) or from Bethyl Labs. Three siRNAs were pooled and protein levels were measured after 48 h of treatment. Western blot band densities were measured using ImageJ software (http://rsbweb.nih.gov/ij/).

### Chromatin immunoprecipitation (ChIP)

Tissues were homogenized using a glass homogenizer and ES cells were collected before, during, and after differentiation. Cells were incubated at room temperature for 15 min in 1% formaldehyde. Crosslinking was stopped by adding 50 µL glycine followed by a 5 min incubation at room temperature and cell lysis as described [66]. Chromatin was sonicated to yield fragments 300–1000 bp in length and was then pre-cleared with protein A agarose beads for 1 h at 4°C. An aliquot of 20 µL was kept to serve as the input fraction. Pre-cleared chromatin was incubated in immunoprecipitation buffer at 4°C overnight using the following antibodies: anti-KDM6A/UTX [7], anti-UTX (Bethyl Labs), anti-H3K27me3 (Millipore), and anti-H3K4me3 (Millipore). Samples were centrifuged at 1200 rpm for 1 min and a small portion of the suspension collected as the unbound fraction. Immunoprecipitated chromatin was collected and serially washed in increasingly stringent salt buffers. After elution, crosslinks were reversed in 5 M NaCl at 65°C overnight. DNA was purified using Qiaquick PCR purification kit (Qiagen) and subjected to PCR according to the following protocol: 95°C for 3 min followed by 35 cycles of 95°C for 30 sec, 56°C for 30 sec and 72°C 30 sec. Samples were incubated at 72°C for 10 min and analyzed by gel electrophoresis. Controls to assay the immunoprecipitation efficiency of KDM6A, H3K4me3, and H3K27me3 antibodies included an active gene (*Kdm5c*) and an inactive gene (*Iqsec2*) (Table S2).

### Quantitative real-time and allele-specific RT–PCR and PCR

For qRT-PCR, total RNA was prepared using the Qiagen RNeasy kit with on-column DNaseI digestion. For cDNA synthesis, 500 ng-1 µg of mRNA was reverse transcribed using the SuperScript First Strand Synthesis system (Invitrogen) according to manufacturer's protocol. Table S2 lists the RT-PCR primers specific for *Rhox6*, *Rhox9*, and *Kdm6a*. Quantitative PCR was performed using a SYBR green master mix (Roche) and a standard curve for each primer pair. Data normalized to the *18s* housekeeping gene were averaged for 2 to 3 separate reactions each assayed in duplicate. For chromatin analyses, ChIP DNA was subjected to real-time PCR using primers listed in Table S2. *Rhox6*R1 and *Rhox9*R1 amplify regions upstream of the transcription start site of their respective gene. *Rhox6/9*R2 amplify regions in the 5′ gene body, and *Rhox6/9*R3 regions towards the 3′ end of both *Rhox6* and *Rhox9*. After normalization to the input fraction, relative enrichment was calculated based on two separate immunoprecipitation reactions each assayed in duplicate. Following PCR amplification, melting curves were used to ensure only a single product was amplified. Western blots were done to confirm sexually dimorphic KDM6A protein levels in female and male ES cells using standard procedures. Briefly, nuclear protein was captured using an anti-KDM6A antibody (Bethyl Labs) using 1∶5000 dilution. Anti-β-ACTIN was used at 1∶10,000 dilution (Sigma) as a loading control. KDM6A protein was detected using HRP conjugated donkey anti-rabbit IgG, and β-ACTIN was detected using HRP conjugated goat anti-mouse IgG.

Allele-specific expression was determined by Sanger sequencing of RT-PCR products and control PCR of genomic DNA using primers listed in Table S2. For *Rhox6*, the SNP (T>G) that distinguishes between the maternal C57BL/6J (Xa) and paternal *M. spretus* (Xi) alleles is at nucleotide position 35180550 (NCBI37/mm9 build). For *Rhox9*, the SNP (T>G) that distinguishes between the maternal C57BL/6J (Xa) and paternal *M. spretus* (Xi) alleles is at nucleotide position 35254278 (NCBI37/mm9 build).

### Expression array analyses

cDNA was hybridized to Affymetrix 1.0 ST and Affymetrix 430 2.0 mouse arrays. Array hybridizations were done at the Microarray Center or at the Center on Human Development and Disability (University of Washington, Seattle WA). The raw data files from the 430 2.0 arrays were analyzed by the Affymetrix software (GCOS 1.1) to produce the data in .CHP format (Excel), while raw data files from the 1.0 ST arrays were analyzed with Affymetrix Expression Console Software (http://www.affymetrix.com). Data was normalized with the RMA method as implemented in the Bioconductor Affymetrix package. Microarray quality control metrics were included according to the manufacturer's recommended guidelines. For analyses of published array data [32], [38], [39], spots were normalized by dividing the signal intensity against the average fluorescent intensity of each array [67]. Expression array data have been deposited in NCBI's GEO database [68] and are accessible through series accession number GSE45034 (http://www.ncbi.nlm.nih.gov/geo/query/acc.cgi?acc=GSE45034).”

### ChIP tiling array analyses

Following ChIP, DNA was amplified by whole genome amplification using the GenomePlex Complete Whole Genome Amplification Kit (Sigma) with modifications previously described [69]. ChIP DNA was lyophilized and re-suspended in 10 µl of water. Library preparation buffer and stabilization buffer were added (2 µl and 1 µl, respectively), and samples incubated at 95°C for 2 min. After addition of library preparation enzyme, samples were incubated in a thermal cycler according to the following protocol: 16°C for 20 min, 24°C for 20 min, 37°C for 20 min, 75°C for 5 min. For amplification of the library, a master mix containing amplification master mix, water, and WGA DNA polymerase was added and samples subjected to 15 cycles of: 95°C for 3 min, 94°C for 15 sec, and 65°C for 5 min. Samples were purified using the Qiaquick PCR purification kit. ChIP and input fractions were labeled according to the standard Nimblegen sample labeling protocol prior to hybridization to HD2 Nimblegen tiling arrays for the entire mouse X chromosome (Roche). Enrichment profiles were generated (Genomics Resource Center, Fred Hutchinson Cancer Research Center, Seattle WA). Peak maps generated by the Nimblescan software consist of significant peaks (FDR score <0.05). Tiling array data have been deposited in NCBI's GEO database [68] and are accessible through series accession number GSE45390 (http://www.ncbi.nlm.nih.gov/geo/query/acc.cgi?acc=GSE45390).”

### Statistics

All p-values shown represent paired two-tailed Student's *t*-tests.

## Supporting Information

Figure S1 *Rhox6* and *9* PCR primer specificity. DNA sequence chromatograms of PCR (gDNA) and RT-PCR (cDNA) products are shown to verify specificity of primers for each gene. Underlined nucleotides differ between *Rhox6* and *9*.
(EPS)

Figure S2 *Rhox6* and *9* expression in female and male embryos and KDM6A occupancy during ES cell differentiation. (A) Average *Rhox6* and *9* expression measured by arrays in 8-cell embryos is higher in 5 female embryos compared to 4 male embryos. Values were divided by the array mean (*p<0.05) (see also Figure 1C). (B) ChIP-tiling arrays for KDM6A occupancy at *Rhox6* and *9* in female PGK12.1 and male WD44 ES cells. KDM6A increases in female ES cells at day 2 of differentiation when expression is high and is very low at day 15 when *Rhox6* and *9* are not expressed (see also Figure 1B). Low levels of KDM6A occupancy are observed in male ES cells at *Rhox6* and *9* whose expression remains low (see also Figure 1B). Raw signal intensities from ChIP-chip represented as log_2_ ratio of ChIP/input. Black bars indicate the position of ChIP-PCR primers R1, R2, and R3 listed in Table S2. Data uploaded to UCSC genome browser (NCBI36/mm8) and nucleotide positions shown.
(EPS)

Figure S3 Confirmation of undifferentiated and differentiated states of female ES cells PGK12.1 by *Xist* analysis. (A) *Xist* expression was measured in undifferentiated (day 0) and differentiated (day 15) female ES cells PGK12.1 by RT-PCR. + indicates RT positive samples and – no RT controls. *β-actin* is used as a positive control. (B) Percentage of interphase nuclei with 0 or 1 *Xist* signals after RNA-FISH on female ES cells PGK12.1 before (day 0) and after differentiation (day 15).
(EPS)

Figure S4 H3K27me3 profile across the *Rhox* cluster after differentiation of female ES cells. ChIP-chip profile reveals high levels of H3K27me3 throughout the *Rhox* gene cluster, reflecting X inactivation at day 15 of differentiation of female ES cells PGK12.1 (see also Figure 2C). Significant ChIP enrichment peaks based on Nimblescan analysis (FDR score <0.05) are shown. Data uploaded to UCSC genome browser (NCBI36/mm8) and nucleotide positions shown at top. * indicates a gap in the tiling array containing no probes.
(EPS)

Figure S5 Specificity of *Kdm6a* knockdown in ES cells. (A) Expression fold change for *Kdm6a*, *Rhox6* and *9* measured by qRT-PCR after *Kdm6a* knockdown in female ES cells PGK12.1 treated for 48 h with two individual siRNAs. siRNA1 and siRNA2 resulted in 63% and 54% knockdown, respectively, and both led to a reduction in *Rhox6* and *9* expression. Expression is shown relative to control levels obtained with scrambled siRNA. (B) Profiles of KDM6A occupancy at *Rhox6* and *9* by ChIP-chip in control treated and *Kdm6a* RNAi treated female ES cells PGK12.1. As expected, KDM6A occupancy is reduced after knockdown. Raw signal intensities from ChIP-chip data represented as log_2_ ratio of ChIP/input. Data uploaded to UCSC genome browser (NCBI36/mm8) and nucleotide positions shown at top. R1, R2, and R3 indicate the position of primers listed in Table S2. (C) No significant changes in H3K4me3 levels at *Rhox6* and *9* as measured by ChIP-qPCR were seen after *Kdm6a* knockdown. (D) *Kdm6a* knockdown causes no changes in expression of known differentiation genes (*Sox2, Pou5f1, Nanog, Stat3, Cd9*) in WD44 and PGK12.1 ES cells. Gene expression measured by array analysis is shown as fold change between knockdown and control levels obtained with scrambled siRNA.
(EPS)

Figure S6 H3K27me3 and H3K4me3 profiles across the *Rhox* cluster in undifferentiated female and male ES cells. H3K27me3 and H3K4me3 enrichment profiles are compared between undifferentiated female ES cells PGK12.1 and male ES cells WD44 at the *Rhox* cluster. Profiles contain only highly significant peaks as determined by Nimblescan software analysis (FDR score <0.05). Only *Rhox6* and *9* (grey boxes) are significantly enriched with both H3K27me3 and H3K4me3 (bivalent) while other *Rhox* genes are contained in silent chromatin (see also Figure 4). Data uploaded to UCSC genome browser (NCBI36/mm8) and nucleotide positions shown at top.
(EPS)

Table S1 *Rhox* gene expression changes in response to *Kdm6a* knockdown. Fold change values (F1-4) were calculated by dividing gene expression values (obtained by expression arrays) for *Kdm6a* RNAi treated (U1-4) samples by control values obtained for siRNA treated samples (C1-4). Average fold change (avg F) represents average expression array results from four individual RNAi experiments using pooled siRNAs. Fold decrease is the inverse value to F. Multiple probesets were averaged and arrays were normalized by their mean. Genes with expression values less than 10% of the mean were discarded. Note, *Rhox5* also appears down-regulated, but it was not further analyzed due to the absence of probes for this gene in tiling arrays.
(XLSX)

Table S2 Primer Sequences. Primer nucleotide sequences for quantitative RT-PCR (qRT-PCR), ChIP-qPCR, and Sanger sequencing are listed.
(XLSX)
